# Oxidative Stress and Inflammation as Targets for Novel Preventive and Therapeutic Approches in Non Communicable Diseases

**DOI:** 10.3390/antiox9040290

**Published:** 2020-03-31

**Authors:** Chiara Nediani, Lisa Giovannelli

**Affiliations:** 1 Department of Experimental and Clinical Biomedical Sciences "Mario Serio", viale Morgagni 50, 50134 Florence, Italy; 2 Department of Neurosciences, Psychology, Drug Research and Child Health (NEUROFARBA), Section of Pharmacology and Toxicology, University of Florence, 50139 Florence, Italy; lisa.giovannelli@unifi.it

As recently reported by the World Health Organization (WHO), Non-Communicable Diseases (NCDs) has been rising over the last century representing the main cause of death and disability for the general population regardless of age, region, or gender [1,2]. NCDs are chronic diseases characterized by long duration and slow progression that account for most ageing-related diseases including cardiovascular and neurodegenerative diseases, cancer, diabetes mellitus and chronic kidney disease. Inflammation, oxidative stress and dysregulated authophagy are common features in NCDs that participate in the progression of these diseases, and may be key targets for the development of novel preventive and therapeutic strategies [3]. This Special Issue consists of 14 articles related to the establishing of specific biomarkers of the features that detail the pathogenesis of the diseases in order to make the correct diagnosis, to evaluate the evolution of the disease and to open up novel strategies of assessment and intervention for the disorders. Kim et al. [4] summarized in a review the recent evidence on the effect of oxidative stress and inflammation on Post-Traumatic Stress Disorder (PTSD), a chronic debilitating condition resulting from trauma exposition. The authors reported that these may be the causes of the neuroinflammatory responses within the brain. In particular, the over-expression of key inflammatory markers, such as IL-6, may be a result of peripheral cytokines, crossing the blood-brain barrier in response to trauma and psychosocial stress, thus leading to neurodegeneration and to neural tissue loss, followed by dysfunction in the respective brain regions. Indeed neuroimaging-based studies have demonstrated that altered inflammatory markers are associated with structural and functional alterations in brain regions that are responsible for the regulation of stress and emotion. Therefore, the different levels of IL-6 in the serum of individuals exposed to trauma according to the source of oxidative stress, suggest that they may be considered as a peripheral marker for PTSD based on trauma type (the presence of TBI or loss of consciousness, or psychosocial trauma). Oxidative stress and chronic inflammation are also responsible for the damage to retinal pigment epithelium (RPE) that contribute to several retinal degenerative diseases, such as age-related macular degeneration or Stargardt disease. Trakkides et al. [5], for the first time, reported that oxidative stress induced an increased expression of the complement regulators (CFH) and properdin and the central complement protein C3 in RPE cells independent of an external complement source. These increases are, on the other hand, associated with inflammasome activation that, subsequently, enhanced secretion of proinflammatory and proangiogenic factors. The authors concluded that complement proteins and receptors derived from RPE were involved in cell homeostasis following oxidative stress and should be considered as targets for treatment development of retinal degeneration. The finding of peripheral biomarkers of oxidative stress correlated with the clinical status of Crohn’s disease (CD) patients, affected by a chronic inflammatory disorder of the intestinal tract, is the aim of the study by Luceri et al. [6]. Indeed these patients are at high risk of post-operative recurrence, so discovering new tools for the assessment of disease activity are needed to prevent long-term complications. In these patients, inflamed bowel tissue and inflammatory cells generate an increase in reactive oxidative species (ROS) that activates a pathogenic cascade, which further exacerbates inflammation and leads to increased oxidative damage to DNA, proteins, and lipids. They found elevated levels of serum advanced oxidation protein product (AOPP), advanced glycated end-products (AGEs), and thiobarbituric acid reactive substances, all markers of oxidative damage, in CD patients with severe relapse, suggesting that these parameters could be evaluated in a prospective as biomarkers for diagnosis or monitoring of CD patients. Therefore, because AOPP and AGEs activate the membrane receptor for advanced glycation end products (RAGE) involved in inflammatory diseases, they hypothesized that AOPP/AGEs activation of RAGE signaling may represent a pathogenic factor in the acute phase of the disease. 

Several approaches have been used to prevent damage from oxidative stress and inflammation. In the context of glomerulonephritis (GN), where heterogenous renal conditions lead end stage renal disease (ESRD), many patients are irresponsive towards the standard immunosuppressive therapies, so alternative interventions to cure or prevent GN-related deterioration is very important from both public health and economic points of view. Increased oxidative stress contributes to the pathogenesis of mesangial proliferative GN. Mesenchymal stem cells (MSCs) of various origins, improve kidney injury because they possess an intrinsic anti-oxidative ability. Chang et al. [7] showed that intrarenal transplantation of hypoxic preconditioned MSCs (HMSC), in an anti-Thy1.1-induced rat glomerulonephritis, was a more effective strategy compared with normal MSC, because HMSC, by activating hypoxic inducible factor-1 /VEGF/Nrf2 (HIF-1 /VEGF/Nrf2) signaling, promoted a further intrinsic anti-oxidative defense preserving anti-oxidant proteins and anti-oxidative responsive element proteins, and, subsequently, reduced glomerular apoptosis, autophagy, and inflammation. Another study by Nuhu et al. [8] showed that the use of i.v. iron concomitantly with antioxidant therapy may improve iron deficiency anemia (IDA) in chronic kidney disease (CKD) without significant impact on renal function or oxidant status. It is well known that IDA can exacerbate mitochondrial dysfunction and enhance oxidative stress in patients with CKD. In an experimental-induced rat model of uremia they found that intravenous iron therapy had a modest impact on iron deficiency anemia in uremic animals, but reduced systemic lipid peroxidation and upregulated systemic GPx activity; in addition it increased mitochondrial maximal respiration and respiratory reserve capacity, suggesting a mitochondrial adaptation or upregulation of their numbers or function.

Many epidemiological and clinical trial studies support that many foods commonly consumed in the Mediterranean diet (MD) contain bioactive compounds with helpful activities which are considered to play significant roles in the prevention and treatment of many diseases [9,10]. Among which olive oil, and in particular extra virgin olive oil (EVOO), is well recognized as one of the healthiest foods in the human diet [11]. The beneficial effects of EVOO is the result of the combination of functional components, such as fatty acid composition (with a high content of oleic acid) and wide minor bioactive constituents, among which polyphenols such as oleuropein from *Olea europaea* L. Several biological properties attributed to oleuropein, providing beneficial effects in the prevention of degenerative diseases, are mainly based on its antioxidant potential [12], but a recent review by Nediani et al. [2] describes how oleuropein has multi-target activity including the anti-inflammatory [13], the anti-amyloid aggregation [14], anti-hypertensive [15], hypoglycemic [16], cardioprotective [17], autophagy inducer [18,19] and anticancer [20], also in combination with standard anti-cancer drugs [21]. In this context, the anti-proliferative and pro-apoptosis activity of oleuropein, in addition to its ability to reduce the glycolytic metabolism of different tumor types [22], it might represent an effective tool for complementary cancer therapy. Due to these biological and biomedical effects of oleuropein, special attention is devoted to the recovery, recycling, and upgrading of food waste, leaves and by-products for its use in agronomic, nutraceutical, and biomedical applications [23]. The beneficial effects of olive and tea leaves or different preparations (e.g., infusions, extracts) have been known since ancient times, and have been used as traditional herbal remedies for the treatment of many diseases, such as diabetes mellitus. Diabetes mellitus is the most prevalent metabolic disorder and is becoming a serious worldwide public health threat because it induces serious complications in several organs. In a wide review Meng et al. [24] summarizes and discusses the effect of tea in the prevention and management of diabetes mellitus and its complications, based on findings from epidemiological, experimental, and clinical studies. Tea, in particular green tea, contains many bioactive compounds, such as catechin like epigallocatechin-3-gallate (EGCG), that seems to have a protective effect on type 1 and 2 diabetes mellitus by protecting pancreatic cells, ameliorating insulin resistance and decreasing hyperglycemia. In addition, tea and its bioactive components, due to their anti-inflammatory and antioxidant potentials, may be used in the prevention of diabetic nephropathy, neuropathy, retinopathy and cardiovascular risks. In addition to tea several other medical plants or natural products have been found to have an anti-diabetic effect with an amelioration of kidney dysfunction due to hyperglycemia-induced renal inflammation. Park et al. [25] showed the protective effect against the diabetic complication of *Lespedeza bicolor* extract (LBE) a perennial deciduous shrub belonging to the Leguminosae family that contains antioxidant phenolic components including genistein, quercetin, and naringin, in an *in vivo* animal diabetic model. They found that LBE supplementation, in addition to decreasing serum fasting blood glucose and glycated hemoglobin A1 at a low dose, improved kidney dysfunction, as demonstrated by a lowered urine albumin-creatinine ratio, while at high dose plasma creatinine, blood urea nitrogen and glomerular hypertrophy appeared to have declined. Furthermore, in T2DM mice a high dose of LBE supplementation significantly attenuated renal hyper-inflammation associated with NLRP3 inflammasome and oxidative stress related to nuclear factor erythroid 2-related factor 2. In the same animal model a low dose of LBE supplementation up-regulated energy metabolism through activation of the adenosine monophosphate kinase (AMPK) /Sirtuin (SIRT)-1 pathway. They concluded that LB supplementation might have beneficial effects to prevent and ameliorate hyperglycemia-induced renal inflammation under diabetic conditions.

T2DM is often associated with obesity (Diabesity) to be defined as the XXI Century epidemic. It is a condition linked to many cardiovascular factor risks such as dyslipidemia, hypertension, and coronary artery disease. Obesity, in particular, may be prevented by an adequate lifestyle and treated, at the preclinical stage with a well-balanced diet, sometimes rich in phenolic compounds. The study of Othman et al. [26] showed the anti-atherogenic effect in high-fat diet (HFD)-induced obese rats of a Malaysian bee bread, that contained, in addition to macronutrient sources and essential minerals and vitamins, also phenolic components such as isorhamnetin, apigenin, caffeic acid, ferulic acid, and kaempferol, which have antioxidant properties. They demonstrated that supplementation of bee bread for 6 weeks reduced the Lee obesity index and levels of total cholesterol, low-density lipoprotein, fatty acid synthase activity and of the atherogenic index, an indicator of high risk to develop CVD. Additionally, the markers of the lipid oxidation process such as oxidised-LDL, and malondialdehyde were significantly decreased, probably due to an increase in aortic antioxidant activities, such as those of superoxide dismutase and glutathione peroxidase. The hypocholesterolemic effect may be attributed to the presence of ferulic and caffeic acids, while kaempherol appears to have an anti-inflammatory effect. These results suggest that bee bread has anti-atherogenic property, partly due to the presence of phenolic compounds which have high antioxidant, anti-inflammatory and hypocholesterolemic properties.

Diets rich in antioxidants may be very important in the prevention and treatment of osteoporosis also. In this context it fit the study of Domazetovic et al. [27] that demonstrated how blueberry juice (BJ) containing a high content of polyphenols, in particular anthocyanins, was able to prevent, in human osteoblast-like SaOS-2 cells, a cellular model to study osteoblast functions, the inhibition of osteogenic differentiation and the mineralization process, due to oxidative stress induced by glutathione depletion. The latter is a condition that mimics a metabolic status of oxidative stress that may occur during estrogen deficiency, as well as in aging and inflammatory diseases, where the decrease in antioxidants leads to accelerated bone loss and, thus, to osteoporosis or osteopenia. The polyphenolic content of BJ exerted its antioxidant action and protection from oxidative stress damage, by upregulating alkaline phosphatase and Runt-related transcription factor 2, markers of the osteoblast differentiation process and regulation of bone remodeling. These factors are, in turn, modulated by activation of SIRT1 expression, a possible molecular target for anti-osteoporotic drugs. These data demonstrated the beneficial effects of BJ rich in polyphenols on bone regeneration, and suggest its use as a dietary supplement for osteoporosis prevention and therapies.

As reported above the search for new antioxidants to be used in therapy and prevention of NCDs often relies on plants. Similar to chemically synthesized molecules, plant bioactive compounds often show multiple activities and specificity towards certain cell types. In this respect, the work by Nieto-Veloza et al. [28] investigates the anti-inflammatory effects of a peptide called BG-4, extracted from a plant of the Cucurbitaceae family, Momordica charantia, known as bitter gourd, cultivated in Asia, Africa, and South America. Previous data indicated a possible anticancer activity of the peptide in the colon. Although active *in vitro* in reducing inflammation and oxidative stress markers in lipopolysaccharide (LPS)-activated mouse macrophages, the peptide was not effective in a model of DSS-induced colitis in mice. Indeed, BG-4 administered to mice at a dose supposed to mimic the concentration effective *in vitro*, aggravated the symptoms. Surprisingly, a reduction of inflammatory cytokines was found in serum but not in colon tissue. Thus, this work points out to the fact that mechanisms such as reduction of NO production, that are protective in simple *in vitro* models, like isolated macrophages, can be toxic in complex *in vivo* systems like colon mucosa, where NO production can exert beneficial effects in enterocytes. Another example of cell specificity is provided by the work of Figueroa-Gonzales and Young [29], who investigated the possible beneficial role of γ-tocoferol in ovarian cancer, based on its ability to both reduce ROS and exert cytotoxicity on cancer cells, making it a good candidate to be used as an adjunctive therapy, helping to preserve proliferating non-cancer cells in the granulosa and avoid post-treatment infertility. They showed that γ-tocoferol was indeed able to reduce chemotherapeutic-generated ROS in ovarian cancer cell lines COV434 and OVCAR, but not in breast cancer cell lines.

Combinations of different compounds or complex matrixes of plant origin can often provide additional advantages compared to single molecules. Fusco et al. [30] showed the positive effects of the combination of melatonin, active on chronobiology, but also a sedative, anesthetic and anti-inflammatory molecule, and folic acid, an antioxidant and immunostimulating agent, in a rat model of fibromyalgia. As for many algic pathologies, an oxidative component has been recently proposed for fibromyalgia, and the addition of an antioxidant compound to different therapeutic strategies appears feasible. The results show that the combination increased the pain threshold, improved motility, reduced lipid and protein oxidation and expression of inflammatory markers in the brain more effectively than the single compounds. Asthma and chronic obstructive pulmonary disease are another research field in which the oxidative component plays an important role, and many plant-derived products have shown a potential beneficial role, which can be, at least in part,attributed to their antioxidant activity. As an example of a complex plant-derived matrix, Kim et al. [31] have investigated the effects of an orally administered dried yeast extract in a mouse model of emphysema induced by passive cigarette smoking. Analyses on animal pulmonary tissue and *in vitro* experiments in the human alveolar cell line A549 showed that the extract was able to reduce cigarette smoking-induced inflammation, apoptosis and proteolytic activity in bronchial and pulmonary tissues, thus reducing emphysema. This effect was accompanied by a reduction in ROS production in the tissue. 
